# Dose development in sinonasal imaging over the last decade – a retrospective patient study

**DOI:** 10.1186/s13005-023-00378-x

**Published:** 2023-07-10

**Authors:** Carsten Hackenbroch, Joachim Rudolf Balthasar Strobel, Kai Johannes Lorenz, Meinrad Beer, Simone Schüle

**Affiliations:** 1 Department of Diagnostic and Interventional Radiology and Neuroradiology, German Armed Forces Hospital Ulm, Ulm, Baden-Wurttemberg, Germany; 2 Department of Otorhinolaryngology and Head and Neck Surgery, German Armed Forces Central Hospital Koblenz, Koblenz, Rhineland-Palatinate, Germany; 3 Department of Radiology, University Hospital of Ulm, Ulm, Baden-Wurttemberg, Germany

**Keywords:** Dose development, Sinonasal imaging, Computed tomography, Chronic sinusitis, Preoperative, Posttraumatic

## Abstract

**Background:**

Computed tomography (CT) has become the primary imaging modality for visualization of the paranasal sinuses. In this retrospective, single center patient study the radiation dose development in the past 12 years in CT imaging of the paranasal sinuses was assessed.

**Methods:**

The computed tomography dose index (CTDI_Vol_) and dose length product (DLP) of a total of 1246 patients (average age: 41 ± 18 years, 361 females, 885 males) were evaluated, who received imaging of the paranasal sinuses either for chronic sinusitis diagnostic, preoperatively or posttraumatically. Scans were performed on three different CT scanners (Somatom Definition AS, Somatom Definition AS+, Somatom Force, all from Siemens Healthineers) and on one CBCT (Morita) ranging from 2010 to 2022. Reconstruction techniques were filtered back projection and three generations of iterative reconstruction (IRIS, SAFIRE, ADMIRE, all from Siemens Healthineers). Group comparisons were performed using either parametrical (ANOVA) or non-parametrical tests (Kruskal-Wallis Test), where applicable.

**Results:**

Over the past 12 years, there was a 73%, 54%, and 66% CTDI_Vol_ reduction and a significant (p < 0.001) 72%, 33%, and 67% DLP reduction in assessing the paranasal sinuses for chronic sinusitis, preoperatively and posttraumatically, respectively.

**Conclusion:**

Technological developments in CT imaging, both hardware and software based, have led to a significant reduction in dose exposure in recent years. Particularly in imaging of the paranasal sinuses, the reduction of radiation exposure is of great interest due to the often young patient age and radiation-sensitive organs in the area of radiation exposure.

## Background

Computed tomography (CT) has become the primary imaging modality for visualization of the paranasal sinuses, whether the clinical indication is to diagnose chronic sinusitis [1, 2] or to visualize the sinuses preoperatively [3] or posttraumatically [4]. Increasing annual CT examination numbers [5, 6] necessitate dose reduction to keep radiation exposure as low as possible, especially since the radiosensitive eye lenses are close to, or, depending on the clinical question, are within the radiation exposure field [7].

Over the last decade numerous dose saving techniques were introduced for CT-examinations [8]. E.g., due to increased computational powers, filtered back projection reconstructions could be replaced by higher quality and dose-saving iterative reconstruction algorithms for CT image reconstruction starting in 2009 [9, 10]. Ever since, continuous improvements of the first-generation iterative reconstruction algorithms have led to even greater dose savings and better image quality [9, 11–13]. Also, CT detectors evolved from gas (‘70s – ‘80s) over solid state (‘90s) to full electronic integrating detectors introduced in the 2010s [8]. With each detector generation, detector efficiency increased, resulting in a lower radiation dose with the same image quality. In 2016 tin prefiltration was introduced by one vendor as another dose saving possibility for high-contrast CT imaging, initially for pulmonary imaging [14, 15] but also for paranasal sinus imaging [16–20]. The tin prefilter removes the softer fraction of the X-ray spectrum and hence, leads to a hardened, more penetrable X-ray spectrum. More photons pass through the patient’s body and reach the detector, resulting in lower image noise at a reduction in dose of approximately 20% [16].

Cone beam CT (CBCT) is considered an alternative, especially for out-clinic patients, for assessing the paranasal sinuses for diagnosing chronic sinusitis and preoperative assessment [21–24]. Radiography has disadvantages because of the difficulty of delineating anatomy due to superimposition, and magnetic resonance imaging is not broadly available in the numbers needed, but is also an alternative, especially for soft tissue imaging.

In this retrospective patient study, the radiation dose of a total of 1246 patients, who received imaging of the paranasal sinuses for chronic sinusitis diagnostics, preoperatively or posttraumatically on three different CT scanners and on CBCT, with special focus on the dose saving potentials of each of the three above mentioned dose saving CT techniques was assessed.

## Methods

### Study population

**Fig. 1:**
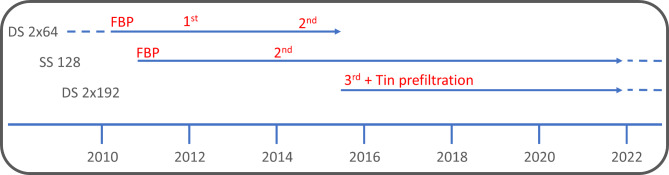
Overview on the used software and hardware. Shown are the three used CT scanners (DS 2 × 64, SS 128, DS 2 × 192), the image reconstruction (FBP, 1st -, 2nd - and 3rd - generation iterative reconstruction) and the tin prefiltering technique and their combination on the time scale from 2010 to 2022 for this retrospective single-center patient study. CT = computed tomography, DS = dual source, FBP = filtered back projection, SS = single source

Ethical approval for this single-center retrospective patient study was waived by the local institutional review board. The scanning parameters (CTDI_Vol_, DLP, tube current, tube voltage) of 45–53 patients per protocol, who underwent CT imaging of the paranasal sinuses, were retrospectively examined. This resulted in a total of 1246 examined patients (average age: 41 ± 18 years, 361 females, 885 males) on three different CT scanners and on one CBCT using different reconstruction algorithms (Filtered back projection (FBP) and iterative reconstruction techniques) with or without tin prefiltration for three different clinical questions (chronic sinusitis, preoperative assessment, posttraumatic assessment) over a period of 12 years from 2010 to 2022 (Fig. 1).

### Image acquisition and reconstruction

Image acquisition and reconstruction took part on three different DECT scanners.

The Somatom Definition AS 64 (Dual Source (DS) 2 × 64) is a dual-source CT scanner of the first generation equipped with a “Multislice Ultra Fast Ceramic” - detector and 2 × 64 detector slices available from 2010 to 2015 at the institution investigated. Images were acquired at 120 kV, tube current between 20 and 100 mAs, pitch between 0.8 and 1 (depending on the year of acquisition and clinical question), and rotation time of 1/s (Table 1). Image reconstruction was performed using FBP, 1st or 2nd generation (Gen.) iterative reconstruction algorithms, with a slice thickness and increment of 1 mm and hard kernels for imaging in the bone window.

The Somatom Definition AS + 128 (Single Source (SS) 128) is a single-source CT scanner equipped with a “Multislice Ultra Fast Ceramic” - detector and 128 detector slices available since 2010 at the institution investigated. Images were acquired at 100 kV, tube current between 25 and 125 mAs (depending on the year of acquisition and clinical question), pitch of 0.8, and rotation time of 1/s. Image reconstruction was performed using FBP or 2nd Gen. iterative reconstruction algorithms, with a slice thickness and increment of 1 mm and hard kernels for imaging in the bone window.

The Somatom Force (DS 2 × 192) is a dual-source CT scanner of the third generation equipped with the more modern “Stellar Infinity” detector, a full electronic integrating detector, and 2 × 192 detector slices available since 2015 at the institution investigated. It was the first CT scanner on the market incorporating a tin prefilter. Images were acquired at 100–120 kV (in parts with tin prefiltration), tube current between 25 and 600 mAs, pitch between 0.6 and 0.8 (depending on the year of acquisition and clinical question), and rotation time of 0.5 to 1/s. Image reconstruction was performed using 3rd Gen. iterative reconstruction algorithms, with a slice thickness between 1 and 2 mm, an increment of 0.75–1 mm and the Hr64 kernel for imaging in the bone window.

The above mentioned hardwares and softwares used for this retrospective, single-center study were produced by Siemens Healthineers AG, Erlangen, Germany.

Additionally, CBCT images taken on a 3D Accuitomo 170 from Morita (J Morita, Kyoto, Japan) during 2015–2017 were evaluated for comparison. Images were acquired with 90 kV and 5-9mA. Image acquisition took 9–17 s and image reconstruction was performed with a slice thickness of 0.5 mm.

**Table 1:** Overview on image acquisition and image reconstruction parameters

		*Image acquisition*												*Image reconstruction*			
**CT Scanner**	**Detector**	**Acquisition year**	**Tin filtration**	**Tube voltage** (kV)	**Tube current** (mAs)		**Collimation** (mm) **x number of slices**		**Pitch**		**Rotation time** (1/s)	**Automatic tube current modulation**		**Reconstruction method**	**Slice thickness** (mm)	**Increment**	**Kernel**
***Chronic sinusitis***																	
Somatom Definition AS 64 (DS 2 × 64)	Ultra fast ceramic	2010–2011	-	120		25		0.6 × 32		1	1	-		FBP	1	1	H60s
		2012–2014	-	120		25		0.6 × 32		1	1	-		IRIS	1	1	J70h
		2015–2016	-	120		20		0.6 × 32		1	1	-		SAFIRE	1	1	H50s
Somatom Definition AS + 128 (SS 128)	Ultra fast ceramic	2011–2013	-	100		35		0.6 × 64		0.8	1	-		FBP	1	1	H60s
		2014–2022	-	100		25		0.6 × 64		0.8	1	-		SAFIRE	1	1	J70h
Somatom Force (DS 2 × 192)	Stellar Infinity	2016	X	100		Ref. 90		0.6 × 96		0.8	0.5	X		ADMIRE	2	1	Hr64d
		2016–2019	X	100		50		0.6 × 96		0.6	0.5	-		ADMIRE	1	1	Hr64d
		2020–2022	X	100		125		0.6 × 192		0.8	1	-		ADMIRE	1	1	Hr64d
3D Accuitomo 170		2015–2017	-	90		5–9		-		-	9–17	-		-	0.5	-	-
***Preoperative***																	
Somatom Definition AS 64 (DS 2 × 64)	Ultra fast ceramic	2010–2011	-	120		30		0.6 × 32		0.9	1	-		FBP	1	1	H60s
		2012–2014	-	120		30		0.6 × 32		0.8	1	-		IRIS	1	1	J70h
		2015–2016	-	120		30		0.6 × 32		0.9	1	-		SAFIRE	1	1	H70h
Somatom Definition AS + 128 (SS 128)	Ultra fast ceramic	2011–2013	-	100		30		0.6 × 64		0.8	1	-		FBP	1	1	H60s
		2014–2022	-	100		25		0.6 × 64		0.8	1	-		SAFIRE	1	1	J70h
Somatom Force (DS 2 × 192)	Stellar Infinity	2016	-	120		Ref. 63		0.6 × 96		0.8	1	X		ADMIRE	1	0.75	Hr64h
		2016–2019	-	100		25		0.6 × 96		0.8	1	-		ADMIRE	1	0.75	Hr64h
		2020–2022	X	100		Ref. 250		0.6 × 192		0.8	1	X		ADMIRE	1	1	Hr64h
***Posttraumatic***																	
Somatom Definition AS 64 (DS 2 × 64)	Ultra fast ceramic	2010–2011	-	120		100		0.6 × 32		1	1	-		FBP	1	1	H60s
		2012–2014	-	120		100		0.6 × 32		1	1	-		IRIS	1	1	J70h
		2015–2016	-	120		70		0.6 × 32		1	1	-		SAFIRE	1	1	H70h
Somatom Definition AS + 128 (SS 128)	Ultra fast ceramic	2011–2013	-	100		125		0.6 × 64		0.8	1	-		FBP	1	1	H60s
		2014–2022	-	100		100		0.6 × 64		0.8	1	-		SAFIRE	1	1	J70h
Somatom Force (DS 2 × 192)	Stellar Infinity	2016	-	120		Ref. 88		0.6 × 96		0.8	1	X		ADMIRE	2	1	Hr64h
		2016–2019	-	100		100		0.6 × 96		0.8	1	-		ADMIRE	1	1	Hr64h
		2020–2022	X	100		600		0.6 × 192		0.8	1	-		ADMIRE	1	1	Hr64h

Overview on technical specification of the three used CT scanners (DS 2 × 64, SS 128, DS 2 × 192) and of the CBCT (3D Accuitomo 170), the image acquisition parameters and image reconstruction parameters of the protocols used in the past 12 years. CT = computed tomography, DS = dual source, FBP = filtered back projection, SS = single source.

### Dose parameter correction

Standard paranasal sinus examinations are calibrated on a 16-cm phantom. From 2016 to 2019 tin prefilter protocols were calibrated on a 32-cm body phantom, as this type of pre-filtering originated from thoracic imaging and the manufacturer did not provide CTDI_Vol_ values calibrated on a 16-cm phantom. Thus, the determined CTDI_Vol_ values in this time period had to be multiplied by a factor of 2.3 according to the manufacturer’s specifications (User manual; Somatom Force, Siemens, Erlangen Germany). Meanwhile, the manufacture provides parameters that are calibrated to a 16-cm phantom.

### Statistics

Data were reported as mean ± standard deviation. Group comparisons were performed using either parametrical (ANOVA) or non-parametrical tests (Kruskal-Wallis Test), where applicable (e.g., examining for normal distribution and equal variance). Dunn’s Test was performed as a post-hoc test. A two-sided significance level of 0.05 was applied. The statistical analysis was performed using SigmaPlot (Version 14.5, Systat Software Inc., San Jose, CF, USA). The graphical representations were performed using PowerPoint (Microsoft, Redmond, United States) and SigmaPlot.

## Results

### Chronic sinusitis

**Fig. 2:**
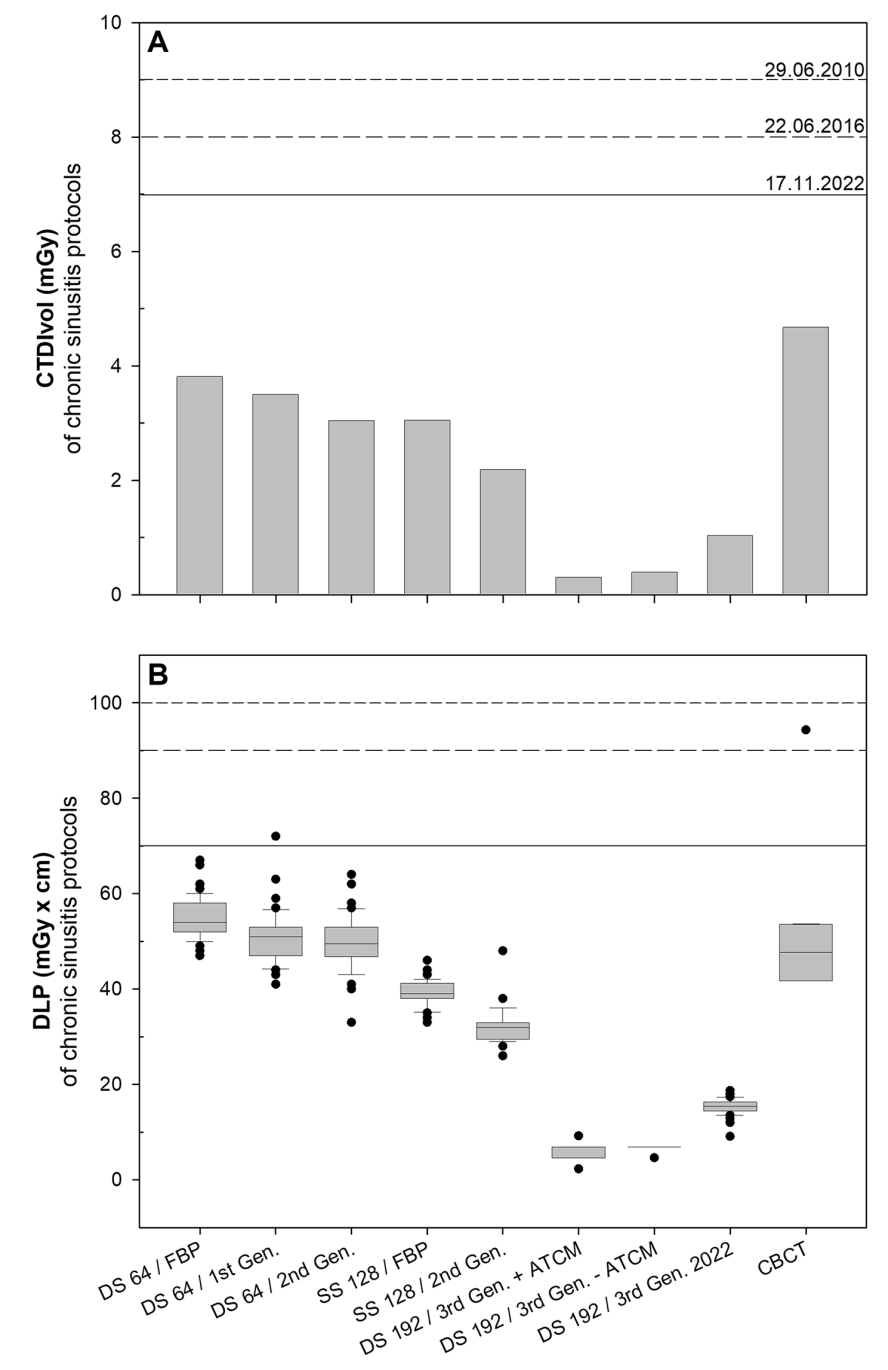
Radiation dose of CT-scans used for diagnosing chronic sinusitis in the past 12 years. Bar chart and Box plot of CTDI_Vol_  **(A)** and DLP **(B)** of protocols (x-scale) used for diagnosing chronic sinusitis. The last bar chart and box plot shows dose values for CBCT. Horizontal lines within boxplots represent medians. Upper and lower whiskers correspond to 1.5 x of the interquartile range. Black dots represent outliers. The protocol name consists out of the Scanner (DS 64, SS 128, DS 192) and the reconstruction method (FBP, 1st, 2nd and 3rd Generation of iterative reconstruction). Since 3rd Generation iterative reconstruction was used for all DS 192 protocols, additional information, such as with or without automatic tube current modulation or the year of acquisition, was added. The short dash, medium dash, and solid horizontal line show the reference values given by the authorities for this CT examination at the time. ATCM = automatic tube current modulation, CBCT = cone beam CT, CT = computed tomography, CTDI_Vol_ = computed tomography dose index, DLP = dose length product, FBP = filtered back projection, Gen. = generation

**Fig. 3:**
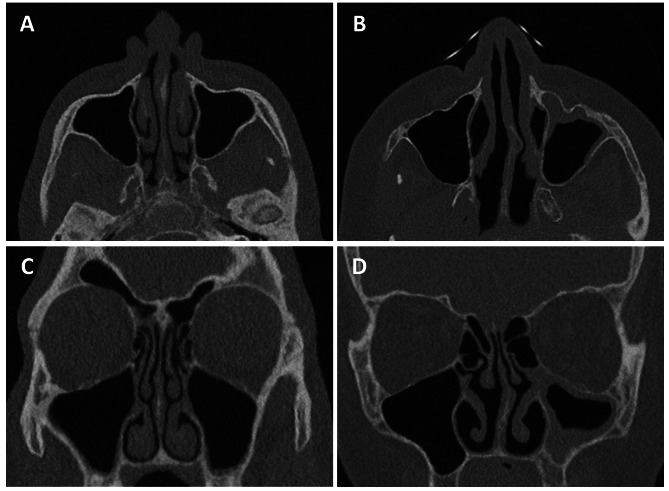
Example CT-images of the paranasal sinus for diagnosing chronic sinusitis. Shown are CT-images in axial and coronal plane in bone window of the DS 192 / 3rd Gen. + ATCM **(A, C)** and of the DS 192 / 3rd Gen. 2022 **(B, D)**. Note the increased image noise and the reduced image quality with poorer delineation of bony structures in A and C

The mean radiation dose measured with CTDI_Vol_ and DLP was below the reference values allowed by the German authorities for the diagnosis of chronic sinusitis during the 12-year observation period (Fig. 2). The German diagnostic reference values were chosen because they provided reference values for each clinical indication studied during this period. Over the last 12 years the CTDI_Vol_ was reduced up to 92% from 3.8 mGy (DS 64 / FBP) to 0.3 mGy (DS 192 / 3rd Gen. + automatic tube current modulation (ATCM)) due to, e.g., tin prefiltration, new detector technologies and the introduction of different generations of iterative reconstructions (Fig. 2A; Table 2). However, while all scans were considered of diagnostic image quality at a CTDI_Vol_ of 0.3 mGy, image quality was not considered satisfactory (Fig. 3), resulting in a recent 3-fold increase in CTDI_Vol_ from 0.3 mGy (DS 192 / 3rd Gen. -ATCM) to 1.0 mGy (DS 192 / 3rd Gen. 2022). Compared to DS 64 / FBP, this still resulted in a 73% reduction in CTDI_Vol_ during the observation period (DS 64 / FBP 3.81 mGy vs. DS 192 / 3rd Gen. 2022 1.03 mGy). This also applies to the corresponding DLP values, which have been significantly (p < 0.001) reduced by 72% over the last 12 years. (55 ± 4 mGy x cm DS 64 / FBP to 15 ± 2 mGy x cm DS 192 / 3rd Gen. 2022, Fig. 2B).

Although mean DLP values continuously decreased, there were no significant differences in DLP values (p ≥ 0.57) between the different reconstruction methods/scanner protocols using the same scanner, except for the comparison between the DS 192 / 3rd Gen. +ATCM and the DS 192 / 3rd Gen. 2022 (p = 0.017) because of the above-mentioned increase in radiation dose with the DS 192 / 3rd Gen. 2022 protocol. Each new CT scanner decreased significantly DLP values (p ≤ 0.003) irrespective of the reconstruction method/scanner protocol, although the DLP values of the DS 192 / 3rd Gen. 2022 and the SS 128 / 2nd Gen. were found to be the only ones that did not differ significantly (p = 1.0). CBCT showed a 1.6- (DLP) to 2.1-fold (CTDI_Vol_) and a 3.5- (DLP) to 4.5-fold (CTDI_Vol_) increased radiation dose compared to the low-dose protocols at that time of the SS 128 /2nd Gen. and DS 192 / 3rd Gen. 2022, respectively. With a CTDI_Vol_ of 4.67 mGy and a DLP of 52 ± 19 mGy x cm the CBCT achieved higher (CTDI_Vol_) or equal (DLP, p = 0.5) radiation doses as the DS 64 / FBP chronic sinusitis protocol.

**Table 2:** Overview on CTDI_Vol_ and DLP values for each scanning protocol for chronic sinusitis diagnostic

	CTDI_Vol_			DLP			
**Scanning protocol**	**Mean** (mGy)	**Reduction in % compared to DS 64 / FBP**		**Mean ± Std. Dev.** (mGy x cm)			**Reduction in % compared to DS 64 / FBP**
DS 64 / FBP	3.8	-		55	±	4	-
DS 64 / 1st Gen.	3.5	8		51	±	6	7
DS 64 / 2nd Gen.	3.0	20		50	±	6	10
SS 128 / FBP	3.1	20		39	±	3	29
SS 128 / 2nd Gen.	2.2	43		32	±	3	42
DS 192 / 3rd Gen. + ATCM	0.3	92		6	±	1	90
DS 192 / 3rd Gen. - ATCM	0.4	90		7	±	1	88
DS 192 / 3rd Gen. 2022	1.0	73		15	±	2	72
CBCT	4.7	+ 23		52	±	19	6

Mean value (± standard deviation) of CTDI_Vol_ and DLP values of protocols used for diagnosing chronic sinusitis in the past 12 years. Additionally, the dose reduction in percent per scan protocol compared the DS 64 / FBP protocol is shown for the CTDI_Vol_ and DLP values. The protocol name consists out of the Scanner (DS 64, SS 128, DS 192) and the reconstruction method (FBP, 1st, 2nd and 3rd Generation of iterative reconstruction). Since 3rd Generation iterative reconstruction was used for all DS 192 protocols, additional information, such as with or without automatic tube current modulation or the year of acquisition, was added. ATCM = automatic tube current modulation, CBCT = cone beam CT, CT = computed tomography, CTDI_Vol_ = computed tomography dose index, DLP = dose length product, FBP = filtered back projection, Gen. = generation.

### Preoperative assessment

**Fig. 4:**
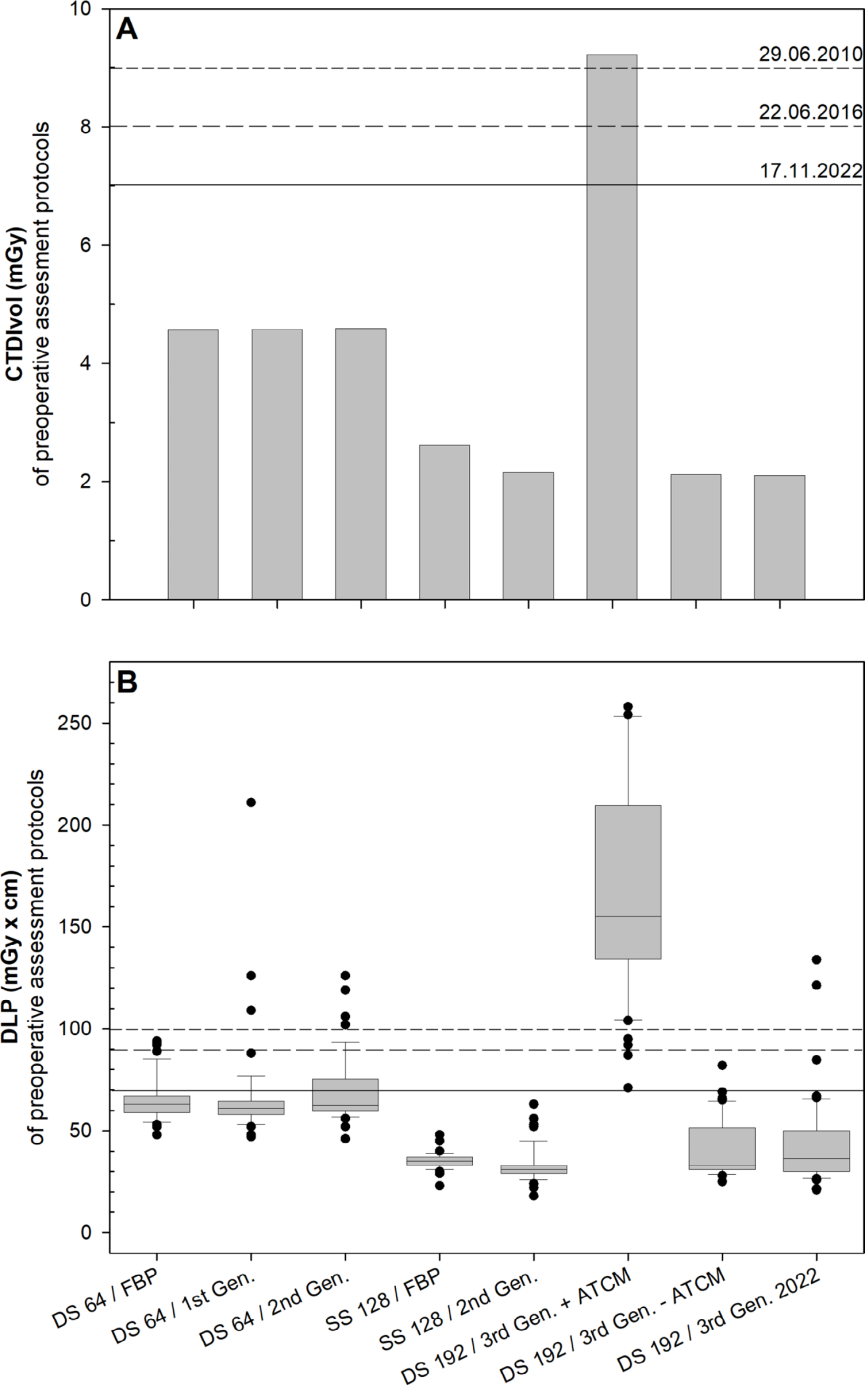
Radiation dose of CT-scans used for preoperative assessment in the past 12 years. Bar chart **(A)** and Box plot **(B)** of CTDI_Vol_  **(A)** and DLP **(B)** of protocols (x-scale) used for preoperative assessment. Horizontal lines within boxplots represent medians. Upper and lower whiskers correspond to 1.5 x of the interquartile range. Black dots represent outliers. The short dash, medium dash, and solid horizontal line show the reference values given by the authorities for this CT examination at the time. ATCM = automatic tube current modulation, CT = computed tomography, CTDI_Vol_ = computed tomography dose index, DLP = dose length product, FBP = filtered back projection, Gen. = generation

The mean radiation dose measured with CTDI_Vol_ and DLP was below the reference values allowed by the authorities for the preoperative assessment of the paranasal sinuses for most protocols during the 12-year observation period (Fig. 4; Table 3). Only the manufacturer-supplied Somatom Force output protocol with ATCM resulted in radiation exposure above the regulatory approved reference level. A correction of the protocol resulted in an overall CTDI_Vol_ reduction (DS 192 / 3rd Gen. +/- ATCM 2.1 mGy) of 54% and a significant DLP reduction (p < 0.001) of 37% (- ATCM, 41 ± 14 mGy x cm) and 33% (+ ATCM, 43 ± 22 mGy x cm) over the last 12 years compared to the DS 64 / FBP protocol (CTDI_Vol_: 4.6 mGy, DLP: 65 mGy x cm) due to, e.g., tin prefiltration, new detector technologies and the introduction different generations of iterative reconstructions.

There were no significant differences in DLP values (p = 1.0) between the different reconstruction methods/scanner protocols using the same scanner, except for the comparison between the DS 192 / 3rd Gen. +ATCM and the DS 192 / 3rd Gen. -ATCM resp. DS 192 / 3rd Gen. 2022 (p < 0.001) because of the above-mentioned increase in radiation dose with the DS 192 / 3rd Gen. +ATCM protocol. The DLP values of the SS 128 and the DS 192 were significantly reduced (p < 0.001) compared to the ones of the DS 64 CT scanner irrespective of the reconstruction method/scanner protocol. However, DLP values of the DS 192 and the SS 128 did not significantly differ (p ≥ 0.331), when the DS 192 / 3rd Gen. + ATCM protocol is disregarded.

**Table 3:** Overview on CTDI_Vol_ and DLP values for each scanning protocol for preoperative assessment

	CTDI_Vol_			DLP			
**Scanning protocol**	**Mean** (mGy)	**Reduction in % compared to DS 64 / FBP**		**Mean ± Std. Dev.** (mGy x cm)			**Reduction in % compared to DS 64 / FBP**
DS 64 / FBP	4.6	-		65	±	10	-
DS 64 / 1st Gen.	4.6	0		67	±	25	+ 3
DS 64 / 2nd Gen.	4.6	+ 1		69	±	17	+ 7
SS 128 / FBP	2.6	43		35	±	4	46
SS 128 / 2nd Gen.	2.2	53		33	±	8	49
DS 192 / 3rd Gen. + ATCM	9.2	+ 102		169	±	57	+ 162
DS 192 / 3rd Gen. - ATCM	2.1	54		41	±	14	36
DS 192 / 3rd Gen. 2022	2.1	54		43	±	22	33

Mean value (± standard deviation) of CTDI_Vol_ and DLP values of protocols used for preoperative assessment in the past 12 years. Additionally, the dose reduction in percent per scan protocol compared the DS 64 / FBP protocol is shown for the CTDI_Vol_ and DLP values. ATCM = automatic tube current modulation, CBCT = cone beam CT, CT = computed tomography, CTDI_Vol_ = computed tomography dose index, DLP = dose length product, FBP = filtered back projection, Gen. = generation.

### Trauma

**Fig. 5:**
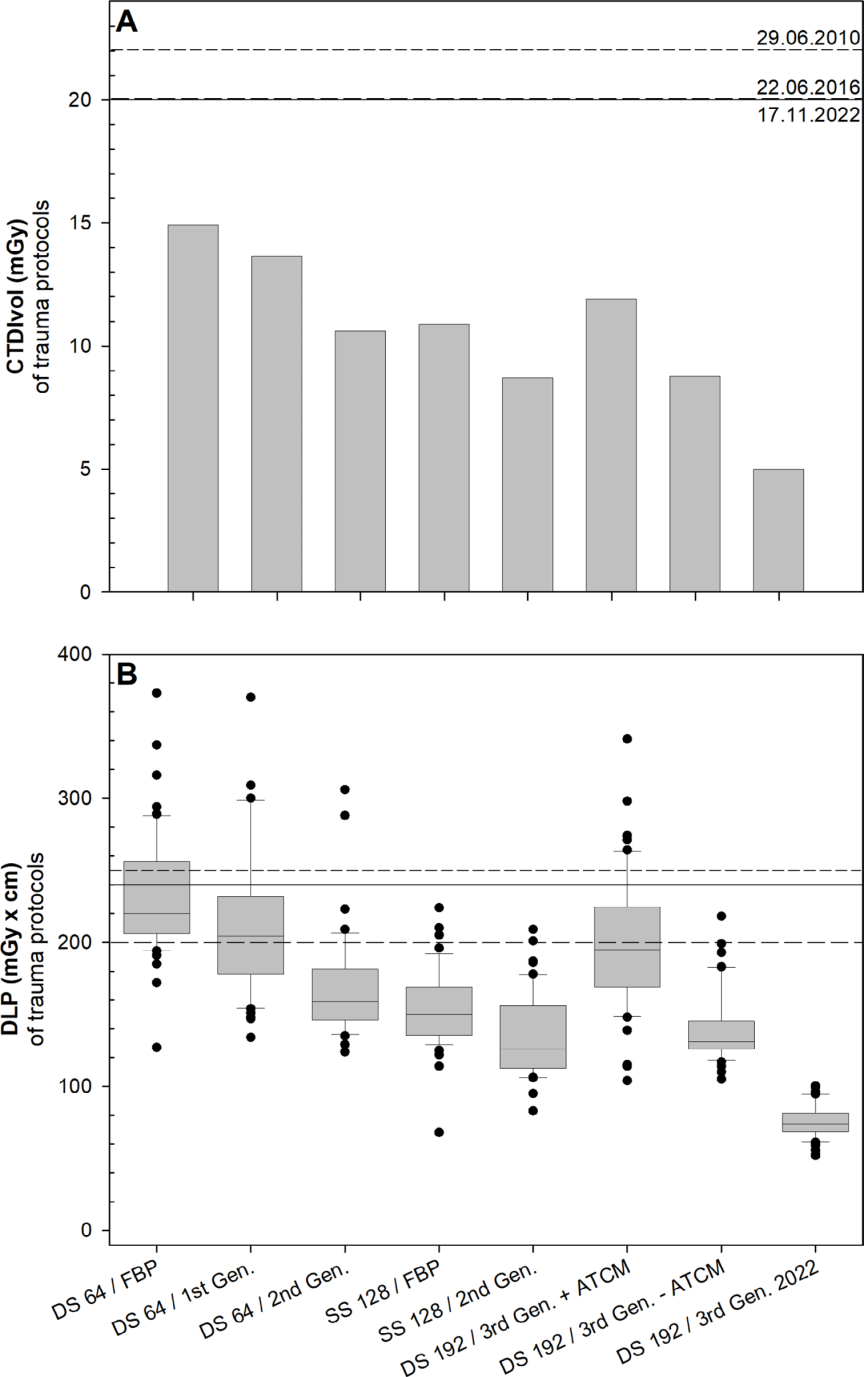
Radiation dose of CT-scans used for trauma assessment in the past 12 years. Bar chart **(A)** and Box plot **(B)** of CTDI_Vol_  **(A)** and DLP **(B)** of protocols (x-scale) used for trauma assessment in the past 12 years. Horizontal lines within boxplots represent medians. Upper and lower whiskers correspond to 1.5 x of the interquartile range. Black dots represent outliers. The short dash, medium dash, and solid horizontal line show the reference values given by the authorities for this CT examination at the time. ATCM = automatic tube current modulation, CT = computed tomography, CTDI_Vol_ = computed tomography dose index, DLP = dose length product, FBP = filtered back projection, Gen. = generation

The mean radiation dose measured with CTDI_Vol_ and DLP was below the reference values allowed by the authorities for trauma diagnostics of the paranasal sinuses during the 12-year observation period (Fig. 5; Table 4). Over the last 12 years the CTDI_Vol_ was reduced up to 66% from 14.9 mGy (DS 64 / FBP) to 5.0 mGy (DS 192 / 3rd Gen. 2022) and DLP values were reduced up to 67% from 232 ± 43 mGy x cm (DS 64 / FBP) to 76 ± 11 mGy x cm (DS 192 / 3rd Gen. 2022) due to, e.g., tin prefiltration, new detector technologies and the introduction of different generations of iterative reconstructions (Fig. 5A; Table 4). As with preoperative assessment protocols, the manufacturer-supplied Somatom Force output protocol resulted in a significant (p < 0.001) increase in DLP from 134 ± 29 mGy x cm (SS 128 / 2nd Gen.) to 199 ± 47 mGy x cm (DS 192 / 3rd Gen. + ATCM) before protocols were corrected and reached the above-mentioned dose savings.

DLP values significantly differed (p ≤ 0.01) when using different reconstruction methods/scanner protocols at the same scanner, except for the comparison between the DS 64 / FBP and the DS 64 / 1st Gen. and the DS 128 / FBP and the DS 128 / 2nd Gen. (p ≥ 0.38). However, mean DLP values also decreased in these two comparisons but did not reach significance. In the cross-scanner comparison DLP values did not significantly differ (p ≥ 0.053) between the DS 64 / 2nd Gen. and the SS 128 / FBP and the DS 192 / 3rd Gen. – ATCM when the DS 192 / 3rd Gen. + ATCM protocol is disregarded. Also, the comparison of the DLP values between the SS 128 protocols and the DS 192 / 3rd Gen -ATCM protocol was not statistically significant (p = 1.0). It was not until the introduction of the tin filter in the DS 192 scanner that the radiation dose was significantly (p ≤ 0.001) reduced compared to the SS 128 protocols.

**Table 4:** Overview on CTDI_Vol_ and DLP values for each scanning protocol for trauma diagnostic

	CTDI_Vol_			DLP			
**Scanning protocol**	**Mean** (mGy)	**Reduction in % compared to DS 64 / FBP**		**Mean ± Std. Dev.** (mGy x cm)			**Reduction in % compared to DS 64 / FBP**
DS 64 / FBP	14.9	-		232	±	43	-
DS 64 / 1st Gen.	13.7	9		218	±	78	6
DS 64 / 2nd Gen.	10.6	29		168	±	36	27
SS 128 / FBP	10.9	27		154	±	27	34
SS 128 / 2nd Gen.	8.7	42		134	±	29	42
DS 192 / 3rd Gen. + ATCM	11.9	20		199	±	47	14
DS 192 / 3rd Gen. - ATCM	8.8	41		140	±	25	40
DS 192 / 3rd Gen. 2022	5.0	66		76	±	11	67

Mean value (± standard deviation) of CTDI_Vol_ and DLP values of protocols used for trauma assessment in the past 12 years. Additionally, the dose reduction in percent per scan protocol compared the DS 64 / FBP protocol is shown for the CTDI_Vol_ and DLP values. ATCM = automatic tube current modulation, CBCT = cone beam CT, CT = computed tomography, CTDI_Vol_ = computed tomography dose index, DLP = dose length product, FBP = filtered back projection, Gen. = generation.

## Discussion

Due to the increasing number of CT examinations per year [5, 6], optimizing the radiation dose in computed tomography is of great interest. Dose reduction techniques are of particular importance in imaging of the paranasal sinuses due to the low patient age and radiation-sensitive organs, which are in or at least near the field of radiation exposure [7]. In addition, CT imaging has become the primary imaging modality for the visualization of the paranasal sinuses by providing three-dimensional imaging of the anatomical structures in high resolution [1, 3, 4, 25]. Over the last decade, innovations, such as improved CT detector technology [26], iterative reconstructions [9, 27–29] and tin prefiltration [16–20] lead to a reduced radiation dose in paranasal sinus imaging. This single-center retrospective patient study evaluated the radiation dose measured by CTDI_Vol_ and DLP over the last 12 years of a total of 1246 patients, who received imaging of the paranasal sinuses for chronic sinusitis diagnostics, preoperatively or posttraumatically on three different CT scanners and one CBCT scanner. Advances in CT imaging over the past decade resulted in a 73%, 54%, and 66% CTDI_Vol_ reduction and a significantly (p < 0.001) 72%, 33%, and 67% DLP reduction for the evaluation of the paranasal sinuses for chronic sinusitis, preoperatively and posttraumatically, respectively.

The currently applied scanning protocols for paranasal sinus imaging are below the reference values allowed by the German authorities [30–32]. Reference values for paranasal sinus imaging have been provided by the German authorities since the beginning of the observation period of this study (2010) and are lower than other international reference values, which is why they were selected for presentation in this study. For example, the United Kingdom introduced the first reference values for the diagnosis of chronic sinusitis in 2022 (CTDI_vol_ 12 mGy), and the United States still has no specific reference values for paranasal sinus imaging [33–35].

A CT scan can be associated with high radiation doses when performed according to the standardized protocols recommended by various CT scanner manufacturers [36, 37], as also shown by the results of this study when the Somatom Force was first used for preoperative assessment and posttraumatic imaging (DS 192 / 3rd Gen. +ATCM) in 2016. It is therefore important to monitor the radiation dose in relation to the image quality regularly and to always critically scrutinize the scanner acquisition settings.

For diagnosing chronic sinusitis, an increased image noise can be acceptable and radiation exposure can thus be reduced during a CT examination without relevant loss of image information [18, 29, 38]. However, preoperatively and especially posttraumatically, low image noise is crucial since the CT image must depict the fine bony structures of the paranasal sinuses, such as the cribriform plate and the lamina papyracea, in great detail [3, 18]. In the evaluated patient cohort, this was shown by a 4-fold (DS 64 / FBP) to 5-fold (DS 192 / 3rd Gen. 2022) increase in radiation dose for posttraumatic assessment and a 1.2-fold (DS 64 / FBP) to 2-fold (DS 192 / 3rd Gen. 2022) increase in radiation dose for preoperative assessment compared with protocols used for the diagnosis of chronic sinusitis.

For chronic sinusitis diagnostics and preoperative assessment, CBCT is also widely used. CBCT has also led to a significant dose reduction in the past decade and provides high spatial resolution, which is one of the main technical advantages of CBCT [21–24]. The most important limitations of CBCT are insufficient soft tissue visualization [22] and the relatively long exposure time with the danger of motion artifacts in restless patients. The CBCT scanner group is also very heterogeneous, with clear qualitative differences between the individual devices (resolution, examination time and dose). Also, in comparison to low-dose or even ultra-low-dose CT [18], the dose exposure associated with CBCT is higher. In the evaluated patient cohort, this was shown by a 1.6-fold (DLP) to 2.1-fold (CTDI_Vol_) and a 3.5-fold (DLP) to 4.5-fold (CTDI_Vol_) increased radiation dose in CBCT compared with low-dose protocols at that time of the SS 128 / 2nd Gen. and DS 192 / 3rd Gen. 2022, respectively.

This study showed an overall 33–74% reduction in dose for imaging of the paranasal sinuses over the last 12 years. However, it is impossible to distinguish the contribution of each technical innovation (detector improvement, iterative reconstructions and their respective generations, and tin filtration) to the described dose reduction, because with the introduction of each technical novelty, additional scanning parameters such as the tube voltage and the tube current were changed. To explore the exact dose saving potentials of each technical innovation, phantom or prospective patient studies with a different, but also more artificial, experimental design are needed, which may introduce its own biases in the results. However, some dose saving effects could be elaborated. (1) For diagnosing sinusitis dose reduction was greatest with the introduction of each new CT scanner. (2) For preoperative assessment radiation dose was lowest at the SS 128 / 2nd Gen. protocol and no further dose reduction could be achieved by the DS 192 CT scanner. (3) In trauma diagnostics, the combination of new scanners and reconstruction techniques, as well as the use of tin filtration, lead to continuous increasing dose savings. Initial studies of radiation dose with photon-counting detectors imply an even greater reduction in radiation dose in the future [39, 40].

## Conclusion

This retrospective patient study, with a total of 1246 included patients, reviewed and demonstrated the dose development in sinonasal imaging over the last decade using state of the art equipment. It showed, that with each detector and iterative reconstruction generation and with the implementation of the tin prefilter, the radiation dose was gradually reduced resulting in two-third to one-third of the radiation dose of 2010. Also, the radiation dose in CBCT is 2–5 fold higher compared to low-dose CT protocols.

## Data Availability

The datasets used and analysed during the current study are available from the corresponding author on reasonable request.

## Abbreviations

ATCM: Automatic tube current modulation
CBCT: Cone beam computed tomography
CT: Computed tomography
CTDI_Vol_: Computed tomography dose index
DLP: Dose length product
DS: Dual source
FBP: Filtered back projection
Gen.: Generation
SS: Single source
